# Permanent education as an inalienable responsibility of health councils: the current scenario in the Unified Health System

**DOI:** 10.6061/clinics/2020/e1443

**Published:** 2020-01-06

**Authors:** Rita de Cássia Costa da Silva, Maykon Anderson Pires de Novais, Paola Zucchi

**Affiliations:** I Programa de Pos Graduacao em Gestao e Informatica em Saude, Escola Paulista de Medicina, Universidade Federal de Sao Paulo, Sao Paulo, SP, BR; II Disciplina de Economia e Gestao em Saude, Escola Paulista de Medicina, Universidade Federal de Sao Paulo, Sao Paulo, SP, BR

**Keywords:** Health Planning Councils, Permanent Education, Continuing Education, Unified Health System, Social Control

## Abstract

**OBJECTIVE:**

To verify whether health councils in Brazil carry out permanent education activities for municipal, state and federal district councilors.

**METHOD:**

This was a cross-sectional study with secondary data collection in the Health Council Monitoring System (Sistema de Acompanhamento dos Conselhos de Saúde - Siacs) from May to August 2017. The Siacs is publicly accessible and available on the internet. It provides data from thousands of health councils throughout Brazil. Analysis and interpretation of the data were based on the literature and the enacted legislation, particularly Resolution 453/2012 and the National Policy of Permanent Education for Social Control in the Unified Health System (Política Nacional de Educação Permanente para o Controle Social no Sistema Único de Saúde).

**RESULTS:**

Despite the fact that Resolution 453/2012 establishes the deliberation, elaboration, support and promotion of permanent education for social control as functions of the councils (in accordance with the guidelines of the National Policy of Permanent Education), approximately 40% of councils do not carry out permanent education.

**CONCLUSIONS:**

It is necessary to strengthen the role of health councils in the elaboration of educational initiatives across the national territory. This includes the allocation of financial resources to increase access to and participation in these initiatives, which would strengthen social control in the Unified Health System. This study emphasizes that the discussion of permanent education is not given sufficient attention in the agendas and routines of health councils. This compromises the effectiveness of councils’ monitoring and deliberation of public health policy.

## INTRODUCTION

The creation of the Unified Health System (SUS) in Brazil dates to the Federal Constitution of 1988, which considered health to be a citizenship right guaranteed by the state via public, universal and decentralized actions and services (1).

Social participation is a guideline of the Brazilian health system, regulated by Law 8142/90, which established councils and health conferences as forms of community participation in the management of the SUS (2).

Health councils, which are the objects of this research, are defined as collegial, deliberative and permanent entities for the social control of public health policy (2). Since 1990, the presence of health councils has been a prerequisite for the transfer of federal resources to all municipalities, states and the Brazilian Federal District.

Among the factors limiting community participation and effective social control in the SUS, the asymmetry of power and knowledge among councilors merits special consideration (3). The concern for the continued education of health councilors was expressed in the National Guidelines for the Qualification of Health Councilors (Diretrizes Nacionais para Capacitação de Conselheiros de Saúde), which were first published in 1999, nine years after the enactment of Law 8142/90. The document shows that even then, the health councils that were spread throughout Brazil had a great demand for information and guidelines for councilor formation and qualification. The text also established the responsibilities of government spheres for the development of qualification activities targeting health councilors (4).

Thus, among the responsibilities of health councils is the development of permanent education activities, which is a condition for the effective exercise of the councils’ deliberative capacities. Thus, access to and participation in permanent education activities is a *sine qua non* condition for councilors to exercise their duties with autonomy, increasing the effectiveness of social control in the SUS. In other words, the qualification of councilors is a potential strategy for effective social control (5).

Several authors have pointed out the need for health councilors—especially user representatives, who account for 50% of council members—to participate in qualification activities that expand their capacity for monitoring and supervising health policy (5-7). Without such activities, the councilor’s roles are severely compromised, and the level of empowerment falls short of what is necessary for the complex task of health policy oversight and monitoring.

Since the establishment of health councils in Brazil, it has been shown that the qualification of councilors is a pressing need. However, this qualification must be permanent and rely on an adequate methodology to provide participants with the ability to assume leading roles in their respective functions. In this sense, both the Ministry of Health and the National Health Council recognize that permanent education is the way to promote effective social control within the scope of the SUS (8-9).

The most recent qualification experience for councilors at the national level was the Qualification Project for Social Control in the SUS, which proposed revisiting the formative process of effectively implementing the National Policy of Permanent Education for Social Control according to a resolution approved by the 15^th^ National Health Conference held in 2015. The project included 68 workshops across several Brazilian states that were held between 2017 and 2018 and provided educational materials available in a free-access digital repository.

Other initiatives that provided courses to train health councilors have taken place in Brazilian states such as Minas Gerais. The state’s health council formed a partnership with the School of Public Health of Minas Gerais to offer courses to municipal councils. However, the courses had a standardized format that was applied to all health regions, despite the state’s large geographic area and its cultural and regional diversity (5).

The need to establish permanent qualification processes to strengthen the effectiveness of health councils in the SUS has also been reiterated by reports from national health conferences (9-11). The responsibility of the National Health Council to formulate—in collaboration with the Ministry of Health—the National Policy of Permanent Education for Social Control in the SUS was clearly defined by the National Guidelines for the Qualification of Health Councilors (4).

The National Policy of Permanent Education for Social Control in the SUS, published in 2006, expanded the conceptual bases of health councilor qualification. The policy included a proper understanding of permanent education, which was clearly seen in its consideration of pedagogical processes aimed at the development of the social subject’s autonomy and agency. These processes aim to ensure the right to health, employing active methodologies that value everyday life (8). The policy can be considered a step forward in the transformation of a simple qualification program into an effective national policy. Its intention was to guide the councils in the development and proposal of qualification strategies encompassing all councilors. The policy also took into consideration the importance of giving due consideration to local and regional particularities, recognizing that based on national policy, state, municipal and federal district councils needed to be involved in developing their own strategies for councilor formation. The document also emphasized the prominent role of state and municipal councils in delegating the responsibility for permanent health education. Moreover, it expressed the view that since permanent education is a strategy for strengthening participation and social control, it should be allocated budgetary and financial resources at the three levels of government. Thus, considering the large geographic area and territorial diversity of Brazil, the need for state and municipal councils to develop local and regional permanent education strategies has been recognized as absolutely imperative.

In the field of health social control, permanent education for councilors is directly related to the realization of the most fundamental principle of the SUS: community participation. In this sense, qualification contributes to the understanding and improvement of the councilor’s role in the community, especially because councils have a deliberative character (6).

Another important factor is that decision-making in the SUS requires topical, up-to-date forms of knowledge that should always be accessible to health councilors (6). However, after almost 30 years since the creation of the SUS and its health councils, the implementation of permanent education processes remains one of the great challenges in achieving genuine social control (7).

Although the permanent formation of health councilors is a relevant topic to the implementation of the SUS, it still occupies a restricted space in academic research. A 2017 survey of Brazilian theses and dissertations published between 2004 and 2013 was unable to find any studies on permanent education and social control (12). This study discusses permanent education as an inalienable task of health councils. Its importance rests on the comprehensiveness of the sample, as well as its use of the National Health Council database, which has rarely been used as a source of research data.

Considering that the development of the Permanent Education Policy for the formation of health councilors in all territories is an inalienable responsibility of health councils, this study presents the results of an extensive survey aimed at verifying whether health councils in Brazil fulfil this responsibility. Therefore, we performed searches in the Health Council Monitoring System (Sistema de Acompanhamento dos Conselhos de Saúde - Siacs) to verify whether health councils are performing their permanent education duties.

## METHODS

This was an observational, transversal study conducted from May to August 2017. Cross-sectional studies allow the identification of the presence or absence of a particular characteristic and are suitable when exposure is relatively constant over time (13).

The study population comprised all municipal, state and Federal District councils in Brazil. The sample included all health councils with up-to-date registration in the Siacs. Sample data were collected from the Siacs through May 2017. Councils that were not registered or up-to-date in the Siacs were excluded. The Siacs was established by the National Health Council in 2012 and contains information on the structure and functioning of the municipal, state and Federal District health councils, forming a unified data network that is available on the internet and publicly accessible. Despite being a constantly updated and information-rich database that allows for the functioning of health councils in Brazil to be characterized, it remains rarely used in academic studies. This situation was one of the motivations for choosing the Siacs as a data source. Another motivation was that the use of the Siacs allowed us to obtain a representative sample of councils with respect to the study variable and increased the visibility of this excellent, easily accessible database that is flexible enough to allow for a variety of data queries.

Secondary data collection was performed directly in the Siacs between May and August 2017. The question “Do you perform health councilor qualification?” summarized the target variable since it refers to one of the responsibilities attributed to the health councils in Resolution 453/2012. The data were organized in a spreadsheet, structured according to the health councils’ geographical distribution (by state) and the presence (‘yes’) or absence (‘no’) of health councilor qualification processes in each council.

Data analysis considered the frequency patterns of yes and no responses. Relative frequency analysis was performed. A result comparison was performed based on the geographical distribution of the health councils registered in the Siacs. The states with greater and lesser frequencies of qualification activities were identified. Data interpretation was carried out based on the literature and was also supported by the reading of government regulations determining council functions and the relationship of councils to permanent education—especially Resolution 453/2012 and the National Policy of Permanent Education for Social Control in the SUS, published in 2006.

The study was approved by the Research Ethics Committee of the Federal University of São Paulo (Universidade Federal de São Paulo), opinion No. 1,424,026.

## RESULTS

The study population consisted of 4,587 (81.46%) councils. The total number of health councils in Brazil is 5,631.

A total of 1,044 councils (19.39%) were excluded, as they lacked complete and up-to-date registration in the Siacs.

The frequency of responses (yes or no) indicated that 2,808 (61.3%) registered councils had permanent education initiatives, while 1,779 (38.7%) did not.

The study also allowed for an assessment of the current council registration status in the Siacs. In addition, we were able to identify the geographical distribution of the councils and the percentage composition of the sample according to each state of the federation (Table 1).

The most representative states—i.e., those with the largest number of registered councils with up-to-date Siacs information—were São Paulo (575), Rio Grande do Sul (472), and Minas Gerais (437). The case of Minas Gerais is particularly interesting. The state has 853 municipalities, which is the largest number of administrative units among the Brazilian states. Considering that as an effect of Law 8.124/90, all municipalities have health councils, it is not surprising that the largest number was found in Minas Gerais. However, only 51.23% of the state’s councils were registered in the Siacs.

Councils that perform permanent education activities were found in all regions, with higher prevalences in the North and Northeast states (Table 2).

When considering the percentage of councils that perform permanent education, a homogeneous distribution was found among Brazilian states, ranging from 45% to 78%. The Federal District was an exception, with a low percentage of only 20%. The highest percentages of councils with no permanent education initiatives were identified in states from the Center-West (Table 2).

As previously explained, the southeastern state of Minas Gerais had the largest number of municipalities and, therefore, the largest number of health councils. However, this superiority did not translate into a high prevalence of permanent education initiatives, with only 52.2% of the councils registered in the Siacs offering qualification initiatives, which was one of the lowest percentages among all states (Table 2).

## DISCUSSION

The study covered a significant percentage of registered councils, which is a testament to the importance of the Siacs as a repository of information and monitoring of health councils.

The State Health Council of Minas Gerais maintains a database of its own, namely, the Database of Minas Gerais Municipal Health Councils (Cadces/MG). This system was implemented in 2013 and collects data similar to those available in the Siacs. This may explain the low percentage of Minas Gerais councils registered in the Siacs. It is our understanding that the prevalence of similar information systems can lead to undesired concurrency, resulting in repeated work for the councils since they must register in two databases instead of just one. In the case of Minas Gerais, this may be causing information to not be recorded in the Siacs.

The participation of health councilors in permanent education activities is recognized as an important strategy for their responsibility to assume a proactive and qualified position in the implementation of public health policy (14). Therefore, it is worrying that a significant percentage of councils distributed throughout the national territory do not promote such resources.

Conducting education activities on a permanent basis is an opportunity to discuss the responsibilities of councilors to formulate and develop health policies (15). Furthermore, these activities provide an opportunity to release updates about SUS legal regulations. These updates are frequently released by means of resolutions, ordinances, and new technical standards. The lack of permanent education activities contributes to unqualified management practices, compromising the exercise of participatory democracy and negatively affecting health councilors’ empowerment (16).

Despite the nationwide educational initiatives described in the literature, this research determined that a significant percentage of councils do not carry out qualification activities per their own admission. This result may be explained by limited access to such activities, possibly related to Brazil’s large territorial area. Even the identified regional workshops, such as the Qualification Project for Social Control in the SUS, are insufficient to ensure the participation of all health councils. These initiatives are still administered vertically, with standardized formats and content that are applied to all councilors, with no consideration for the particularities of the territories and health councils. Moreover, the number of vacancies in these initiatives is insufficient to ensure the participation of the large number of health councilors spread throughout the national territory. For this reason, these initiatives are designed to train ‘multiplier’ agents who are responsible for passing on the workshops’ discussions and transmitted knowledge to their peers, thus promoting permanent education activities in the daily routines of health councils. The results obtained in this study, however, indicate that multiplication activities require better follow-up to ensure that the relevant information and knowledge is passed on to all councilors in all health councils. As such, there is an ever greater need for the development of the competency established in Resolution 453/2012 of the National Health Council—that is, councils’ responsibility to deliberate, elaborate, support and promote permanent education in favor of social control in accordance with the National Guidelines and the National Policy of Permanent Education for Social Control in the SUS (17).

Councils that do not perform qualification activities may adopt the perspective of continuing education, which is periodic and based on traditional, outmoded teaching methodologies (18). In this sense, a negative response to the question of whether the council provided health councilor education may reflect a lack of recognition for educational spaces other than formal courses as possible areas for problem-solving and collective construction. The daily life of the councils, which is based on previous knowledge and new, collectively produced meanings, is itself a formative space because processes of permanent education cannot occur without a primary, proactive role by the subjects of a collective (19).

The lack of formative activities is also an obstacle to the very functioning of the councils since it results in a lack of fundamental knowledge and ignorance of the subjects discussed in plenary meetings. These are difficulties that can be minimized only by councils’ adoption of permanent qualification routines. In fact, a previous Brazilian study showed greater capacity for intervention during meetings, as well as better articulation with social movements, among councilors who had undergone qualification activities (15). Conversely, another study showed that councilors perceived permanent education activities to be insufficient (20).

Although legal norms formally adopt the concept of permanent education, the practice is still tied to the conception of training, which is comprised of formal, sporadic, and temporary activities. On the other hand, we cannot be certain that councils claiming to have permanent education activities as part of their routine clearly perceive these activities as a tool for strengthening participatory democracy (8). Even the National Health Council itself, when proposing this question to the councils via the Siacs, adopted the concept of training, which, from theoretical and methodological points of view, is situated in the field of continuing education. The use of the training concept may reinforce the idea of formal, sporadically held courses (21). Hegemonic and fragmented practices persist in health training processes, including in councils. This is a challenge that has to be overcome since it has been widely demonstrated that traditional education practices do not allow for collective construction. Collective construction must be based on listening and dialogue, which are indispensable prerogatives of social control (19).

It is necessary, then, to recognize new spaces for discussion and adopt participatory methodologies that favor engaged and proactive health councils, as proposed by the constructivist approach to the teaching-learning process (21). In addition to expanding access to formative activities, it is also necessary to adopt methodologies that foster critical thinking and value everyday life as a learning space (22).

Formative initiatives targeting national and state-based health councilors should increasingly consider the profile of competencies and educational needs of social actors, focusing more on promoting autonomy and dynamism in the learning process and less on the transmission of predetermined contents that are easily accessible by a person with a critical and proactive stance. Thus, rather than merely transmitting content, formative processes targeting councilors should contribute to subjects’ development of the capacity to learn; in other words, formative processes should be a way of ‘learning how to learn’ to promote the ability to reflect on previous knowledge to construct new hypotheses and explanations for daily experiences (21). In this sense, the observed homogeneous distribution of councils conducting formative activities in the national territory can be a favorable condition for stimulating the exchange of knowledge about successful experiences and the expansion of formative activities, with the councils themselves being units for the promotion of permanent education.

Permanent education is a potential means for the transformation of work practices and processes since it strengthens the action-reflection-action dynamic (22). The lack of formative activities and/or permanent education activities identified in our research limits the effective exercise of democratic social control. Corroborating our findings, a study carried out in 2010 in Brazil identified a lack of qualification as a difficulty for councilors’ exercise of their roles in supervising and deliberating over public policies (3). Health councils can provide qualification activities for councilors through continuous initiatives that involve sharing of knowledge in pre-established spaces such as council and committee meetings and that can foster a collaborative learning perspective through exchange among peers (23). This will lead to the adoption of strategies that foster critical and reflexive reasoning beyond temporary educational activities aimed at the unilateral transmission of knowledge. These strategies have already been introduced in the SUS by Ordinance 198/2004 (22,24).

The findings of Ouverney et al. (6) also corroborate ours. The authors emphasize the need for councils to take the lead in developing formative initiatives, taking authorship of demands and organizing activities based on their formative needs.

## CONCLUSION

Although the research question did not address the councils’ understanding of formative processes, this study has shown that councils’ inalienable responsibility regarding permanent education activities—established in Resolution 453/2012 and reaffirmed by the National Policy of Permanent Education for Social Control in the SUS—is not being fulfilled by all health councils. Although permanent education has been established as a national policy for more than 10 years, the study found that it is still not a universal priority for Brazilian health councils. This situation directly interferes with the quality of the councils’ performance, jeopardizing their role in formulating and controlling health policy implementation.

Permanent education for social control is still not a well-studied field and deserves future research to elucidate how the implementation of educational processes in the councils has taken place so far and what challenges have to be overcome so that all health councils may assume the primary task of councilor formation.

This article demonstrated that, despite the existence of several documents and a wide discussion about the need to adopt permanent education strategies for qualification for social control actions in the SUS, many councils are still not carrying out these actions. We have shown that the inalienable responsibility of health councils for the development of the Permanent Education Policy for the Qualification of Health Councilors is still not fulfilled by approximately 40% of the councils registered in the Siacs.

National formative initiatives targeting councilors are based on previously defined content that is necessary for the satisfactory execution of council functions. However, initiatives that take into account the specific demands and needs of different territories must be strengthened through the allocation of financial resources, among other measures. In addition to offering technical content, permanent education activities should strengthen councilors’ problem-solving capacities. These activities should be based on the day-to-day work of the councilors since the obstacles to democratic exercise in Brazil are still significant.

The identification of regions and states where there is greater access to formative activities may guide further actions and investments in the qualification of health councilors. It is our conclusion that SUS managing bodies must encourage proactive leadership by the councils in regard to assuming responsibility for the formation of their members; this is indispensable to ensuring effective social control of public health policy.

## AUTHOR CONTRIBUTIONS

Silva RCC contributed to the research conception and design, results discussion, writing, and approval of the final version of the manuscript. Novais MAP contributed to the discussion, critical review of content, and approval of the final version of the manuscript. Zucchi P contributed to the research conception and design, results discussion, critical review of content, and approval of the final version of the manuscript.

## Figures and Tables

**Table 1:** Number of councils per state.

STATE	Frequency (N)	%
Acre	23	0.5
Alagoas	97	2.1
Amazonas	62	1.3
Amapá	17	0.4
Bahia	338	7.4
Ceará	256	5.6
Federal District	5	0.1
Espírito Santo	75	1.6
Goiás	211	4.6
Maranhão	135	2.9
Minas Gerais	437	9.5
Mato Grosso do Sul	75	1.6
Mato Grosso	101	2.2
Pará	108	2.3
Paraíba	182	4.0
Pernambuco	146	3.2
Piauí	150	3.4
Paraná	375	8.2
Rio de Janeiro	92	2.0
Rio Grande do Norte	165	3.6
Rondônia	40	0.9
Roraima	10	0.2
Rio Grande do Sul	472	10.3
Santa Catarina	273	5.9
Sergipe	76	1.7
São Paulo	575	12.5
Tocantins	91	2.0
Grand total	**4587**	**100.0**

**Table 2:** Councilor qualification according to the geographical distribution of the councils.

		Councilor qualification				
		NO		YES		Total
REGION	STATE	N	%	N	%	N
North	Acre	5	21.7	18	78.3	23
	Amapá	4	23.5	13	76.5	17
	Amazonas	27	43.5	35	56.5	62
	Pará	37	34.3	71	65.7	108
	Rondônia	9	22.5	31	77.5	40
	Roraima	5	50.0	5	50.0	10
	Tocantins	39	42.9	52	57.1	91
Northeast	Alagoas	27	27.8	70	72.2	97
	Bahia	167	49.4	171	50.6	338
	Ceará	62	24.2	194	75.8	256
	Maranhão	63	46.6	72	53.4	135
	Paraíba	96	52.7	86	47.3	182
	Pernambuco	51	34.9	95	65.1	146
	Piauí	82	54.7	68	45.3	150
	Rio Grande do Norte	68	41.2	97	58.8	165
	Sergipe	29	38.2	47	61.8	76
Center-West	Federal District	4	80.0	1	20.0	5
	Goiás	98	46.4	113	53.6	211
	Mato Grosso	49	48.5	52	51.5	101
	Mato Grosso do Sul	20	26.6	55	73.4	75
Southeast	Espírito Santo	21	28.0	54	72.0	75
	Minas Gerais	209	47.8	228	52.2	437
	Rio de Janeiro	29	31.5	63	68.5	92
	São Paulo	235	40.9	340	59.1	575
South	Paraná	93	24.8	282	75.2	375
	Rio Grande do Sul	154	32.6	318	67.4	472
	Santa Catarina	96	35.2	177	64.8	273
Total		**1779**		**2808**		**4587**
